# Chemical Evaluation, Phytotoxic Potential, and In Silico Study of Essential Oils from Leaves of *Guatteria schomburgkiana* Mart. and *Xylopia frutescens* Aubl. (Annonaceae) from the Brazilian Amazon

**DOI:** 10.3390/molecules28062633

**Published:** 2023-03-14

**Authors:** Ângelo Antônio Barbosa de Moraes, Márcia Moraes Cascaes, Lidiane Diniz do Nascimento, Celeste de Jesus Pereira Franco, Oberdan Oliveira Ferreira, Tainá Oliveira dos Anjos, Himani Karakoti, Ravendra Kumar, Antônio Pedro da Silva Souza-Filho, Mozaniel Santana de Oliveira, Eloisa Helena de Aguiar Andrade

**Affiliations:** 1 Faculdade de Engenharia Química, Instituto de Tecnologia, Universidade Federal do Pará, Rua Augusto Corrêa, 01, Guamá, Belem 66075-900, PA, Brazil; 2 Laboratório Adolpho Ducke, Coordenação de Botânica, Museu Paraense Emílio Goeldi, Av. Perimetral, 1901, Terra Firme, Belem 66077-830, PA, Brazil; 3 Programa de Pós-Graduação em Química, Universidade Federal do Pará, Rua Augusto Corrêa, 01, Guamá, Belem 66075-900, PA, Brazil; 4 Programa de Pós-Graduação em Biotecnologia e Biodiversidade—Rede BIONORTE, Universidade Federal do Pará, Rua Augusto Corrêa, 01, Guamá, Belem 66075-900, PA, Brazil; 5 Programa de Pós-Graduação em Botânica Tropical, Universidade Federal Rural da Amazônia, Avenida Tancredo Neves, 2501, Belem 66077-830, PA, Brazil; 6 Department of Chemistry, College of Basic Sciences and Humanities, G.B. Pant University of Agriculture and Technology, Pantnagar 263145, Uttarakhand, India; 7 Embrapa Amazônia Oriental, Av. Dr. Enéas Pinheiro, Marco, Belem 66095-903, PA, Brazil

**Keywords:** Amazonian essential oils, spathulenol, caryophyllene oxide, α-pinene, β-pinene, phytotoxicity, molecular modeling

## Abstract

The essential oils (EOs) of *Guatteria schomburgkiana* (Gsch) and *Xylopia frutescens* (Xfru) (Annonaceae) were obtained by hydrodistillation, and their chemical composition was evaluated by gas chromatography-mass spectrometry (GC/MS). Herbicide activity was measured by analyzing the seed germination percentage and root and hypocotyl elongation of two invasive species: *Mimosa pudica* and *Senna obtusifolia*. The highest yield was obtained for the EO of Xfru (1.06%). The chemical composition of Gsch was characterized by the presence of the oxygenated sesquiterpenes spathulenol (22.40%) and caryophyllene oxide (14.70%). Regarding the EO of Xfru, the hydrocarbon monoterpenes α-pinene (35.73%) and β-pinene (18.90%) were the components identified with the highest concentrations. The germination of seeds of *S*. *obtusifolia* (13.33 ± 5.77%) showed higher resistance than that of seeds of *M*. *pudica* (86.67 ± 5.77%). *S. obtusifolia* was also more sensitive to the EO of Xfru in terms of radicle (55.22 ± 2.72%) and hypocotyl (71.12 ± 3.80%) elongation, while *M*. *pudica* showed greater sensitivity to the EO of Gsch. To screen the herbicidal activity, the molecular docking study of the major and potent compounds was performed against 4-hydroxyphenylpyruvate dioxygenase (HPPD) protein. Results showed good binding affinities and attributed the strongest inhibitory activity to δ-cadinene for the target protein. This work contributes to the study of the herbicidal properties of the EOs of species of Annonaceae from the Amazon region.

## 1. Introduction

Synthetic herbicides have been widely used in agriculture for the control of invasive plants. However, their excessive and inappropriate use has resulted in resistant weeds, as well as problems such as environmental pollution, food contamination, and threat to human and animal health [1]. Thus, the search for natural herbicides with low toxicity and high efficiency has increased [2].

Essential oils are natural products derived from the secondary metabolism of aromatic plants, which are strong candidates in the development of natural herbicides, as there are records in the literature that report the phytotoxic potential of these volatile oils against invasive species, as observed in species of the family Anonnaceae [3].

The EOs of Annonaceae species have shown diverse biological potentials, as reported in several studies in the literature [4,5,6,7,8]. In recent years, research on EOs has evolved and received greater attention from the academic community and industry sectors [9,10,11,12,13]. The species of Annonaceae from the Amazon may be a renewable source for the production of secondary metabolites with several properties of scientific and industrial interest; such a utilization scheme could generate employment, income, and development for the region [14,15].

Compounds in aromatic and medicinal plants have been widely studied not only for antioxidant potential [16], but also as possible phytotoxic agents against weeds because they play significant roles in soil rhizosphere signaling, chemical ecology, and plant defense; such applications can ensure the survival of other plant species of socioeconomic and scientific interest through processes related to physiological growth, cellular development, and root elongation [17,18,19]. 

The species *Mimosa pudica* L. and *Senna obtusifolia* (L.) Irwin and Barneby are invasive plants found in the Amazon that prevent the growth of pastureland, directly harming agriculture and livestock in the region [20,21]. *S. obtusifolia* also causes toxicity in cattle [22]. Recent studies investigated the phytotoxic potential of EOs on these two invasive species with the aim of benefiting local agroindustry [23,24,25]. 

*Guatteria schomburgkiana* Mart. (Gsch) is one of the species of Annonaceae found in Brazil and in the states of Pará, Amazonas, Mato Grosso, Maranhão, Paraíba, and Pernambuco [26,27]. The extracts of its leaves and bark are characterized by the presence of alkaloids derived from puterine and are used by traditional Amazonian communities as antimalarial agents [28].

*Xylopia frutescens* Aubl. (Xfru) is a species distributed throughout almost all of Latin America [29]. In Brazil, it is found in all states of the southeast Region and in almost all states of the north and northeast Region, in addition to Mato Grosso and Goiás. It is commonly known as red embira and is used in traditional medicine for the treatment of rheumatism and as a bladder stimulant [30]. Phytochemical studies indicate the presence of oxygenated diterpenes, especially kaurenoic acid, in its extracts [31].

Despite the large number of species of Annonaceae present in the Amazon and Pará, studies on the chemical composition and herbicidal activities of the EOs of individuals belonging to this family are still scarce, especially regarding Gsch and Xfru. The objective of this study was to evaluate the chemical composition and herbicidal activity (phytotoxicity) of the EOs of the species Gsch and Xfru against two invasive species common in the Amazon region (*M. pudica* L. and *S. obtusifolia*).

## 2. Results and Discussion

### 2.1. Yield and Phytochemical Profile of Essential Oils

**Figure 1:**
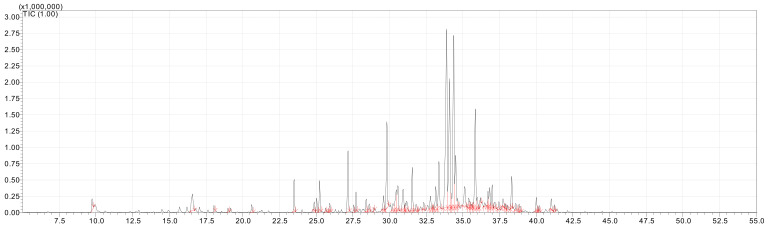
Ion chromatogram (GC/MS) of the essential oil of *Guatteria schomburgkiana*.

**Figure 2:**
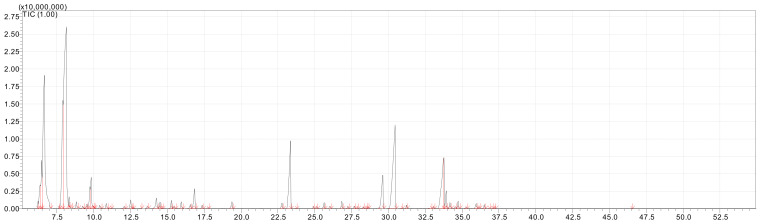
Ion chromatogram (GC/MS) of the essential oil of *Xylopia frutescens*.

Table 1 presents the results for the yield and chemical profile of the EOs of the assessed species of Annonaceae. Ion chromatograms are shown in Figure 1 and Figure 2.

The yields of the EOs of Gsch and Xfru were 0.36% and 1.06%, respectively. Trigo et al. [33] found a yield of 0.20% for the EO from the leaves of Gsch. The yield for the present samples is higher than that reported in the literature. Cascaes et al. [3] and Shakri et al. [34] obtained yields of 1.50% and 0.15%, respectively, for EO from the leaves of Xfru. These values indicate that the yield of the EO of Xfru in the present study is lower than that found by Cascaes et al. [3] and considerably higher than that reported by Shakri et al. [34]. Such differences may be associated with several factors, such as the geographical location, seasonal and circadian cycles of the plants, stage of maturation of the specimens, and soil conditions [35,36,37,38].

In the EO of Gsch, 84.13% of the components were identified, totaling 37 volatile constituents. The chemical profile of the sample was characterized by oxygenated sesquiterpenes (53.75%) and hydrocarbon sesquiterpenes (25.15%), with a predominance of spathulenol (15.42%), fokienol (11.70%), caryophyllene oxide (9.65%), muurola-4,10(14)-dien-1-β-ol (6.49%), and germacrene D (5.26%).

According to Trigo et al. [33], the EO of the leaves of a specimen of Gsch collected in the municipality of Magalhães Barata, Pará, Brazil, contained spathulenol (22.40%), caryophyllene oxide (14.70%), *p*-cymene (8.70%), and dilapiole (5.00%) as the main volatile constituents. The spathulenol and caryophyllene oxide levels found by the authors were higher than those in the present study. In the present samples, the hydrocarbon monoterpene *p*-cymene presented a very low concentration (0.77%), and the phenylpropanoid dilapiole was not identified.

The EOs of other species of the genus *Guatteria* are also characterized by high levels of spathulenol, as identified for *G. juruensis* (77.50%), *G. elliptica* (34.10–53.90%), *G. poeppigiana* (53.00%), *G. australis* (40.29%), and *G. megalophylla* (27.76%) [39,40,41,42]. Caryophyllene oxide is the major component of the EOs of *G. microcalyx* (44.20%), *G. ferruginea* (40.13%) and *G. latifolia* (31.45%) [41,42]. Costa et al. [43] identified high levels of oxygenated sesquiterpenes in the EO of *G. friesiana*, especially the combination of β- and α-eudesmol (58.10%) and γ-eudesmol (16.80%).

The major constituent is used as a flavoring agent in various products of the food and cosmetics industries [44] and has several properties reported in the literature, such as antibacterial, antioxidant, anti-inflammatory, antimycobacterial, antiproliferative, cytotoxic, and insecticidal properties [45,46,47,48,49,50,51]. Gyrdymova and Rubtsova [52] state that caryophyllene oxide is an important compound for organic and medicinal chemistry due to its specific structure that favors the production of several other biologically active structures with analgesic, anti-inflammatory, sedative, cytotoxic, and leishmanicidal potentials [53,54,55,56].

Regarding fokienol, EOs with high concentrations of this oxygenated sesquiterpene have antiviral, antioxidant, anti-inflammatory, antitumor, and gastric activities [57]. Muurola-4,10(14)-dien-1-β-ol has been found in EOs with antimicrobial and larvicidal properties [58,59], and germacrene D exhibits cytotoxic, antimicrobial and anti-inflammatory activities and moderate antioxidant potential [60,61,62].

Regarding the EO of Xfru, 99.23% of the sample was identified, corresponding to 54 chemical constituents. Hydrocarbon monoterpenes were predominant in the sample (58.70%), and the isomers β-pinene (35.73%) and α-pinene (18.90%) were the major volatile constituents, followed by the sesquiterpenes bicyclogermacrene (11.07%), spathulenol (7.37%), and δ-elemene (6.55%). Cascaes et al. [3] evaluated the chemical composition of a specimen of Xfru collected in Magalhães Barata, Pará, Brazil, in March 2018. According to the authors, β-pinene and α-pinene were also the main components, with contents of 25.95% and 20.84%, respectively.

Nascimento et al. [63] analyzed the chemical profile of a specimen collected in the Serra de Itabaiana National Park, Sergipe, Brazil. According to these authors, the sesquiterpene hydrocarbons bicyclogermacrene (23.23%), germacrene D (21.16%), and €-caryophyllene (17.53%) were the main chemical components of the sample and may have been responsible for the repellent and larvicidal activities of the EO. Shakri et al. [34] found that the EO of a specimen of Xfru originating in Malaysia was characterized by hydrocarbon sesquiterpenes (56.80%) and oxygenated sesquiterpenes (39.30%), mainly bicyclogermacrene (22.80 ± 0.20%), germacrene D (14.20% ± 0.30%), elemol (12.80% ± 0.20%), and guaiol (12.80% ± 0.20%). The results of these authors show that the samples have a different chemotype from the specimens collected in Magalhães Barata, Pará, Brazil.

The compounds β-pinene and α-pinene were also identified in the EO of *Xylopia aethiopica*, according to the analysis by Tegang et al. [64] with contents of 32.16 ± 3.69% and 7.39 ± 1.69%, respectively. Kouame et al. [65] found that the EOs of the leaves and fruits of a sample of *X. aethopica* were also characterized by β-pinene (16.01–20.50%) and α-pinene (10.39–17.77%). Costa et al. [66] identified high levels of α-pinene (28.00 ± 1.50%) and β-pinene (5.50 ± 0.10%) in the EO of the fruit of *X. laevigata*. Maia et al. [67] stated that α- and β-pinene are the major components of the EO of *X. cayennensis*, with contents of 29.20% and 16.50%, respectively. Peres et al. [68] indicated that the EOs of the leaves and fruits of *X. aromatica* have high levels of α-pinene (8.23–35.40%) and β-pinene (7.75–22.51%).

The bicyclic monoterpenes α- and β-pinene are well represented in several EOs [69,70]. They are known for their antibiotic resistance, anticoagulant, antitumor, antimicrobial, antimalarial, anti-inflammatory, leishmanicidal, anticonvulsant, anticancer, genotoxic, and cytotoxic activities and for their analgesic, gastroprotective, anxiolytic, cytoprotective and neuroprotective effects; they are used as components of drugs for the treatment of kidney and liver diseases [71,72,73,74,75,76].

(*E*)-Caryophyllene exhibits analgesic, anticarcinogenic, anti-inflammatory, anticonvulsant, myorelaxant, antidepressant and antitumor activities [77,78]. In addition, (*E*)-caryophyllene is emitted by plants as a defensive agent against possible threats to survival [79]. Bicyclogermacrene has been associated with larvicidal and antiviral effects against SARS-CoV-2, which has caused the COVID-19 pandemic [80]. According to Lu et al. [81] δ-elemene has anticancer activity against NCI-H292 lung cancer cells. Fabri et al. [82] showed that this sesquiterpene induces cellular apoptosis in adenocarcinoma cells.

### 2.2. Herbicidal Activity of Essential Oils

The results of the herbicidal activity of the EOs are presented in Table 2. The EOs of Gsch and Xfru showed the same percentage of germination inhibition for *M. pudica* and *S. obtusifolia*, with equivalent values of 86.67 ± 5.77%, and 13.33 ± 5.77%, respectively. In general, *M. pudica* has shown sensitivity to compounds present in EOs, as observed for the EO of *Syzygium aromaticum* [83].

Regarding the inhibitory effects on root elongation, the EO of Xfru showed the highest inhibition potential at 55.22 ± 2.72% (*M. pudica)*, while for *S. obtusifolia*, the value was 60.43 ± 4.63%. The present results are different from those observed by Franco et al. [24] for the EO from *Calycolpus goetheanus* specimen C, which showed inhibition values of 33.33 ± 5.77% and 6.67 ± 5.77% for *M. pudica* and *S. obtusifolia*, respectively.

The effects of the EOs of the two Annonaceae species on hypocotyl elongation showed an intense effect against germination in *M. pudica*. The inhibition values were 71.12 ± 3.80% for the EO of Xfru and 70.95 ± 4.37% for the EO of Gsch. *S. obtusifolia* showed lower susceptibility to the effects of the EOs, with intensity levels of 51.38 ± 1.05% and 51.13 ± 4.50% for the EOs of Xfru and Gsch, respectively. In the literature, a herbicide is considered to have a satisfactory effect when the inhibition percentage is greater than 50% [84,85], as was observed for hypocotyl elongation in all cases analyzed in the present study.

The phytotoxic potential presented in the samples may be related to oxygenated monoterpene compounds and hydrocarbon sequiterpenes that characterized the chemical profile of essential oils [86]. Reports in the literature on the herbicidal activity of EOs from species of Annonaceae are scarce. However, Yoshida et al. [87] indicated that the EO of *Unonopsis guatterioides* has potential as a herbicide in the germination, growth, and development of monocot (*Allium cepa*) and dicot (*Lactuca sativa*) plants. In addition, the authors emphasize that the EO contains components present in the EOs of Xfru and Gsch, such as α-copaene (15.70%), bicyclogermacrene (15.70%), €-caryophyllene (15.70%), α-humulene (9.00%), *allo*-aromadendrene (8.40%), and spathulenol (7.30%).

The inhibitory effects on germination, radicle elongation and hypocotyl elongation obtained in this study may be associated with the presence of oxygenated compounds in the EO samples, according to the trend in the literature [88,89,90]. In addition, the synergistic and/or antagonistic effects of the volatile components in the samples may contribute to their biological activities [91,92,93,94]. According to Galán-Pérez et al. [95], monoterpenes are potent inhibitors of the germination and growth of several plant species. In addition, the authors note that oxygenated monoterpenes have greater water solubility and inhibitory activity than hydrocarbon monoterpenes.

Regarding the major components of the EOs, Fernandes-Silva et al. [96] showed that the volatile concentrate of a sample of Brazilian green propolis contained spathulenol as one of the main components (23.40%). According to the authors, the volatile concentrate influenced both the seed germination and seedling growth of lettuce. Cândido et al. [97] indicated that the EO of the stem of *Croton doctoris* contained caryophyllene oxide (24.50%) as the major component. The authors emphasized that this EO exhibited herbicidal activity on seed germination and on lettuce radicle and hypocotyl growth.

Chowan et al. [98] stated that the hydrocarbon monoterpene β-pinene, a major constituent of Xfru, also has potential as a herbicide in the early growth of rice; this compound promotes biochemical changes by increasing the activity of the enzymes peroxidase and polyphenol oxidase and the content of macromolecules (proteins and carbohydrates), in addition to reducing the total chlorophyll content of rice coleoptiles, suggesting a negative impact on photosynthesis. According to Caputo et al. [99], α-pinene has inhibitory effects on lettuce germination and rootlet growth and is one of the main factors responsible for the phytotoxicity of *Rosmarinus officinalis* EO.

### 2.3. Molecular Docking

The use of computational methods has been increased to test the bioactive potential of the target compounds. In silico molecular docking helps to understand bioactive compounds’ behavior in the HPPD protein’s binding sites [100]. In this sense, in the docking study, the binding energies of the major constituents of the EOs ranged from −7.8 to −5.2 kcal/mol, indicating moderate to good inhibition of the enzyme (Table 3). δ-Cadinene and bicyclogermacrene fit strongly in the enzyme pocket. δ-Cadinene exhibited the best binding affinity with the HPPD protein (−7.8 kcal/mol). The best docked pose with the lowest binding energy was selected by interpolating among multiple docked poses. The best docked pose of δ-cadinene exhibited two pi-alkyl interactions, one pi-sigma interaction and other van der Waals interactions with amino acid residues in HPPD such as Phe A:424, Phe A:381, and His A:308.

**Figure 3:**
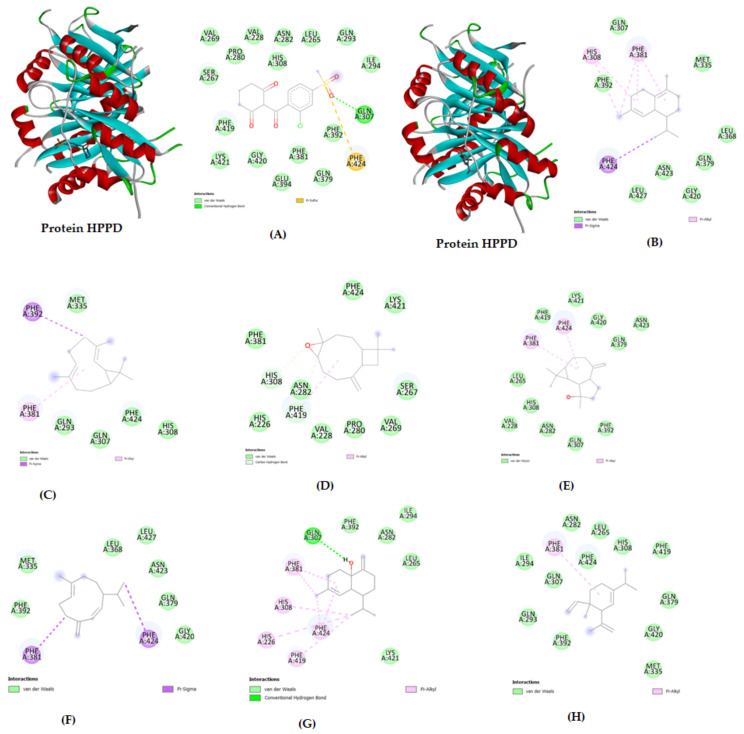
Docked conformations of molecules in the binding cavity of HPPD (PDB: 6J63) with the lowest binding energies. The formed complexes are (**A**) sulcotrione in the pocket of HPPD, (**B**) δ-cadinene in the pocket of HPPD, (**C**) 6J63-bicyclogermacrene, (**D**) 6J63-caryophyllene oxide, (**E**) 6J63-spathulenol, (**F**) 6J63-germacrene D, (**G**) 6J63-muurola-4,10(14)-dien-1-β-ol, and (**H**) 6J63-δ-elemene.

For comparison purposes, a docking study of sulcotrione was also performed, as sulcotrione is a known inhibitor of HPPD. The binding energy of sulcotrione complexed with HPPD was found to be −7.6 kcal/mol with pi-sulfur, hydrogen bond, and van der Waals interactions with amino acid residues. The docking results indicated that the major compounds interacted favorably with the receptors, mostly via hydrophobic interactions, and ligand recognition analysis revealed that the compounds can be good phytotoxic agents. The interactions of compounds with the lowest binding energy (highest docking score) and their 2D interactions are illustrated in Figure 3A–D. The 2D interactions of other compounds with good docking scores are illustrated in Figure 3E–H.

### 2.4. In Silico ADMET Study

**Figure 4:**
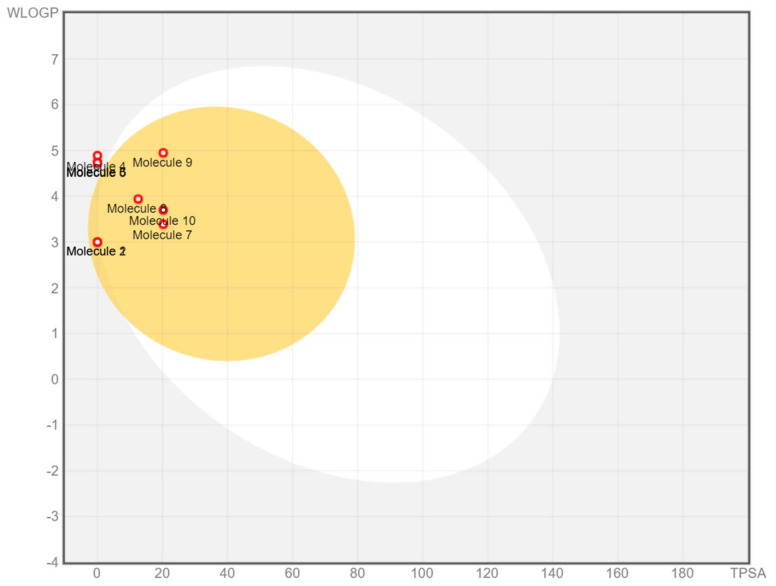
Boiled-egg graph of selected molecules: 1: α-pinene, 2: β-pinene, 3: δ-elemene, 4: germacrene D, 5: bicyclogermacrene, 6: δ-cadinene 7: spathulenol, 8: caryophyllene oxide, 9: fokienol, 10: muurola-4,10(14)-dien-1-β-ol.

According to the in silico results obtained with ADME Swiss software, all of the selected volatile compounds followed the collective laws of Lipinski [101], Egan [102], and Veber [103] that determine the drug-like properties of compounds. For all of the chosen compounds, the bioavailability score was 0.55, indicating higher bioactivity. All the compounds shared a total polar surface area (TPSA) of 0.00 Å except for spathulenol, caryophyllene oxide, fokienol, and muurola-4,10(14)-dien-1-β-ol, which showed good brain penetration and good lipophilicity behavior, as shown in Table 4. No P-glycoprotein (P-gp) substrate was observed, indicating effective intestinal absorption of the compounds. The compounds that were predicted not to cross the blood-brain barrier (BBB) were δ-elemene, germacrene D, bicyclogermacrene, and δ-cadinene. Some of the compounds interacted mainly with two isoenzymes of the cytochrome (CYP) family, namely, CYP2C19 and CYP2C9, suggesting their efficiency yet minimal toxicity. The drug-like properties of selected compounds from Gsch and Xfru were also represented by the boiled-egg prediction graph in which the compounds present in the yellow zone can permeate the BBB (Figure 4). The toxicity parameters of selected volatiles were predicted using the ProTox II web server (Table 5). All the selected compounds were predicted not to be hepatotoxic, carcinogenic, cytotoxic, immunotoxic, or mutagenic, except for germacrene D and caryophyllene oxide, which were immunotoxic. The LD_50_ values were also calculated to ensure the safety of the selected compounds, as shown in Table 5. Compounds with LD_50_ values greater than 2000 mg/kg are considered safe for biological administration and as potential drugs. 

In addition, the phytotoxic agents/herbicides are supplied to effluence and ailments, ranging from skin annoyance to tumors. Compounds with a known or different mode of action that is non-hazardous or harmful to the applicator, animals, or the environment are needed. Chemical libraries are commonly designed and chosen based on the physicochemical properties of the compounds [104]. These characteristics properties are mostly related to chemical compounds’ “absorption, distribution, metabolism, and excretion (ADME)” properties, which have a significant impact on their pharmacokinetic profiles. The distributions of physicochemical character and structural characteristics have been studied for many pesticides, including various subsets of herbicides [105]. These studies have led to the discovery of herbicide-likeness rules, which are analogous to drug-likeness rules that make it simpler to design and produce new herbicide or phytotoxic agents. Different software and online tools are used to predict the ADME properties of compounds.

## 3. Materials and Methods

### 3.1. Collection and Processing of Botanical Material

The botanical material was collected in the municipality of Magalhães Barata, Pará, Brazil (latitude: 0°47′53″ South, longitude: 47°36′10″ West), in May 2019, following conventional procedures. The specimens were incorporated into the Herbarium of the Museu Paraense Emílio Goeldi (Emílio Goeldi Museum) *Guatteria schomburgkiana* Mart (voucher code (*MG237512*)), and Xylopia frutescens Aubl. voucher code (*MG237492*). Then, the material was dried, ground, homogenized, weighed, and subjected to hydrodistillation.

### 3.2. Obtaining Essential Oils by Hydrodistillation

The processed botanical material was subjected to hydrodistillation using a modified Clevenger-type apparatus for 3 h. After the end of distillation, the EOs were centrifuged for 5 min at 3000 rpm, dehydrated with anhydrous sodium sulfate, and centrifuged again under the same conditions. Then, they were stored in amber glass ampoules and kept in a refrigerator at 5 °C.

### 3.3. Yield of Essential Oils

The moisture content of the materials was determined using a moisture analyzer (ID50, Marte Científica, Sao Paulo, Brazil) via infrared spectroscopy. The EO yield was calculated on a moisture-free basis (MFB), as proposed by Santos et al. [106].

### 3.4. Chemical Evaluation of Essential Oils

The chemical profile of the EOs was analyzed by GC/MS in a SHIMADZU QP Plus-2010 system equipped with a DB-5MS silica capillary column (30 m × 0.25 mm; 0.25 m film thickness) under the following operating conditions: carrier gas: helium, at a linear velocity of 36.5 cm·s^−1^; type of injection: splitless (2 µL of oil in 0.5 mL of hexane); injector temperature: 250 °C; temperature program: 60–250 °C, with a gradient of 3 °C·min^−1^; temperature of the ion source and other parts: 220 °C.

The quadrupole filter swept in the range from 39 to 500 daltons·s^−1^. Ionization was performed in electron impact mode at 70 eV. The identification of volatile components was based on the linear retention index (RI) and on the mass spectral fragmentation pattern by comparing the data with authentic samples in database libraries (NIST-11, FFNSC-2) and in the literature (Adams) [32]. The RIs were obtained using the homologous series of *n*-alkanes.

### 3.5. Phytotoxic Protocols

The phytotoxicity bioassays were performed based on the protocols by Batista et al. [107] and Gurgel et al. [108]. Initially, for germination, test solutions consisting of 5 mL of each EO and 50 mL of hexane were prepared. Subsequently, each 9.0 cm diameter Petri dish was lined with a sheet of qualitative filter paper. The plates of the control group received only distilled water, while the others received 3.0 mL of the test solution. After evaporation of the solvent, 20 seeds were placed on each plate, constituting an experimental plot. The test solutions were added only once, at the beginning of the bioassays; in the other steps, only distilled water was added when necessary. The study was conducted in a germination chamber with a constant temperature of 25 °C and a 12 h photoperiod. The germination bioassay was performed in triplicate.

Seed germination was monitored over five-day periods, with daily counts. A seed with a root extension equal to or greater than 2.00 mm was considered to be germinated [33].

For the radicle and hypocotyl development bioassay, the seeds were initially placed in an acrylic gerbox (11 × 11 × 4 cm) with two sterilized sheets of blotting paper as substrate. Forty seeds were distributed in each box and allowed to germinate for three days in a germination chamber with a constant temperature of 25 °C and a 24 h photoperiod. After this time, the plates of the control group received only distilled water, while the other plates received 3.0 mL of the test solution. The test solutions were added only once, at the beginning of the bioassays, and from then on, only distilled water was added when necessary; the bioassay was performed in triplicate. At the end of the five-day growth period, the lengths of the radicle and hypocotyl were measured [109].

### 3.6. Molecular Docking Studies

The molecular docking study of selected volatiles from Gsch and Xfru was carried out on the 4-hydroxyphenylpyruvate dioxygenase (HPPD) protein. HPPD was selected as the target protein because it is the molecular target for compounds with postemergence herbicidal activity. In plants, the inhibition of HPPD results in the depletion of carotenoids, and the absence of chloroplast development in emerging foliar tissues results in necrosis and death [110,111]. The X-ray crystal structure of the HPPD protein (PDB: 6J63) was downloaded from the RCSB protein data bank. Compounds with higher percent content were selected, and their 3D structures were downloaded from the PubChem database (https://pubchem.ncbi.nlm.nih.gov/ accessed on 1 December 2023) in SDF format. Molecular docking studies of the major constituents on the HPPD protein were performed using PyRx software with the Vina Wizard tool to determine the binding energy and the various ligand–receptor interactions responsible for the herbicidal activity [112]. For comparison purposes, a docking study of HPPD was also performed with its known inhibitor, sulcotrione [110]. Biovia Discovery Studio was used for the 2D and 3D visualization of docking poses.

### 3.7. In Silico ADMET Study

The absorption, distribution, metabolism, and excretion (ADME) properties of the selected compounds were estimated using the SwissADME online server (http://www.swissadme.ch/ accessed on 2 December 2023), and the toxicity parameters of the selected compounds were predicted using the ProTox II web server (http://tox.charite.de/protox_II accessed on 1 December 2023). The structures of the selected compounds were imported into the webserver in SMILES format, and the drug-like properties, pharmacokinetic properties, and toxicity parameters (organ toxicity, oral toxicity, and toxicological endpoints) were predicted as per the developed protocol [113].

### 3.8. Statistical Analysis

The results for the herbicidal activity of the EOs were subjected to analysis of variance (ANOVA), and their means were compared using Tukey’s test (at a significance level of *p* < 0.05).

## 4. Conclusions

The essential oils studied show that they are rich in compounds of the monoterpene and sesquiterpene class. The essential oils demonstrate that they have a phytotoxic potential in different intensities of responses, which depends on the receptor specie of plant weeds. However, more studies should be conducted to analyze the phytotoxic potential of the EOs using different concentrations. In addition, by the analysis of ligand interaction with the proteins, the molecular docking study suggested that the compounds from the EOs can be good herbicidal agents. The ADMET analysis also revealed the safety of most of the major compounds in the EOs. This study contributes to the knowledge of the chemical composition of the EOs of species of the Annonaceae family that originate from the aromatic flora of the Amazon region and their potential as herbicides against *M. pudica* and *S. obtusifolia*, invasive species very common in the Amazon.

## Figures and Tables

**Table 1:** Chemical constituents of the essential oils of *Guatteria schomburgkiana* (Gsch) and *Xylopia frutescens* (Xfru).

Species				Gsch	Xfru
Yield (%)				0.31	1.06
RT	RI_L_	RI_C_	Chemical Constituent	Area (%) *	
6.375	924	927	α-Thujene		2.21
6.667	932	938	α-Pinene		18.90
7.692	969	970	Sabinene		0.14
8.158	974	986	β-Pinene		35.73
8.333	988	991	Myrcene		0.27
8.825	1002	1006	α-Phellandrene		0.35
9.258	1014	1017	α-Terpinene		0.15
9.542	1020	1029	*p*-Cymene	0.77	0.31
9.750	1025	1029	Sylvestrene		1.42
9.833	1026	1031	1,8-Cineole		1.73
9.542	1044	1052	(*E*)-β-Cymene		0.18
9.750	1054	1057	γ-Terpinene		0.30
9.833	1065	1065	*cis*-Sabinene hydrate		0.03
12.517	1086	1088	Terpinolene		0.13
12.667	1095	1105	Linalool		0.59
13.242	1100	1108	*n*-Nonanal		0.03
13.650	1112	1116	*trans*-Thujone		0.02
14.267	1126	1125	α-Campholenal		0.12
14.500	1139	1139	*trans*-Pinocarveol		0.90
14.658	1137	1144	*cis*-Verbenol		0.41
15.283	1140	1148	*trans*-Verbenol		0.03
15.442	1160	1162	Pinocarvone		0.48
15.950	1166	1172	*p*-Mentha-1,5-dien-8-ol		0.15
16.575	1174	1183	Terpinen-4-ol		0.42
16.850	1183	1192	Cryptone	1.73	
17.367	1186	1196	α-Terpineol		0.30
17.750	1194	1202	Myrthenol		1.44
19.383	1204	1215	Verbenone		0.18
23.367	1215	1224	*trans*-Carveol		0.06
23.742	1238	1245	Cumin aldehyde	0.17	
24.917	1249	1254	Geraniol		0.42
25.150	1279	1281	Felandral	0.43	
26.050	1335	1342	δ-Elemene		6.55
26.842	1346	1346	α-Terpinyl acetate	1.67	
27.258	1348	1359	α-Cubebene		0.02
28.417	1369	1377	Cyclosativene	0.37	
28.633	1373	1381	α-Ylangene	0.73	
28.950	1379	1383	Geranyl acetate		0.06
29.608	1374	1386	α-Copaene	1.62	11
30.233	1387	1395	β-Bourbonene	0.19	
30.475	1389	1402	β-Elemene		0.36
30.575	1403	1413	Methyl eugenol	0.40	0.18
30.942	1417	1434	(*E*)-Caryophyllene	3.72	42
31.083	1430	1442	β-Copaene	0,34	0.11
31.825	1434	1444	γ-Elemene	0.94	
31.942	1439	1451	Aromadendrene	0.69	15
32.342	1454	1456	Isogermacrene D		0.05
32.800	1445	1461	Myltayl-4(12)-ene		3
32.992	1452	1466	α-Humulene	0.46	
33.375	1458	1471	*allo*-Aromadendrene	0.11	4
33.900	1478	1480	γ-Muurolene	0.78	
34.108	1484	1496	Germacrene D	5.26	279
34.208	1492	1501	*cis*-β-Guayene	0.61	
34.383	1500	1509	α-Muurolene	1.69	
34.517	1500	1514	Bicyclogermacrene	1.79	11.07
34.650	1509	1518	α-Bulnesene	1.87	
35.400	1513	1528	γ-Cadinene	0.71	
35.508	1522	1537	δ-Cadinene	2.39	0.17
35.708	1545	1540	Selina-3,7(11)-diene	0.51	
35.858	1544	1555	α-Calacorene	0.37	
35.992	1562	1562	*epi*-Longipinanol		0.01
36.833	1577	1579	Spathulenol	15.42	7.37
37.008	1582	1592	Caryophyllene oxide	9.65	
37.167	1590	1592	Globulol		1.07
37.467	1592	1598	Viridiflorol		0.39
37.750	1596	1063	Fokienol	11.7	
37.925	1600	1606	Rosifoliol	3.61	0.13
38.050	1608	1625	Humulene epoxide II	1.77	
38.342	1608	1629	β-Atlantol	0.48	
38.617	1618	1632	Junenol	0.31	
38.842	1629	1634	Eremoligenol		0.40
38.950	1630	1634	Muurola-4,10(14)-dien-1-β-ol	6.49	
40.017	1639	1636	Epoxide-*allo*-aromadendrene		0.05
40.108	1652	1669	α-Cadinol		0.22
40.208	1674	1673	Allo-himachalol		0.04
41.042	1676	1676	Guaia-3,10(14)-dien-11-ol		0.04
			Hydrocarbon monoterpenes	0.77	58.70
			Oxygenated monoterpenes	0.43	6.59
			Hydrocarbon sesquiterpenes	25.15	21.87
			Oxygenated sesquiterpenes	53.75	9.72
			Phenylpropanoids	3.97	0.90
			Hydrocarbons		1.45
Total				84.13	99.23

Note: RT = retention time; RI_L_ = retention index in the literature (Adams) [32]; RI_C_ = retention index calculated from a homologous series of *n*-alkanes (C8-C40) in a DB5-MS column. * Relative area (%) calculated based on peak area.

**Table 2:** Herbicidal activity (%) of the essential oils of species of Annonaceae.

*X. frutescens*	Mean (%)
Germination (*Mimosa pudica*)	86.67 ± 5.77 ^b^
Radicle (*M. pudica*)	55.22 ± 2.72 ^a^
Hypocotyl (*M. pudica*)	71.12 ± 3.80 ^b^
Germination (*Senna obtusifolia*)	13.33 ± 5.77 ^c^
Radicle (*S. obtusifolia*)	60.43 ± 4.63 ^a^
Hypocotyl (*S. obtusifolia*)	51.38 ± 1.05 ^a^
*G. schomburgkiana*	Mean (%)
Germination (*Mimosa pudica*)	86.67 ± 5.77 ^a^
Radicle (*M. pudica*)	50.00 ± 1.17 ^a^
Hypocotyl (*M. pudica*)	70.95 ± 4.37 ^b^
Germination (*Senna obtusifolia*)	13.33 ± 5.77 ^c^
Radicle (*S. obtusifolia*)	46.05 ± 4.97 ^a^
Hypocotyl (*S. obtusifolia*)	51.13 ± 4.50 ^a^

Values are expressed as the mean and standard deviation (n = 3) of herbicide activity. Different letters indicate that the samples are significantly different.

**Table 3:** Binding energy (−kcal/mol) of selected phytocompounds from Gsch and Xfru complexed with 6J63.

S. No.	Compounds (PubChem CID)	Binding Energy (−kcal/mol)
	α-Pinene (6654)	−5.4
	β-Pinene (14896)	−5.5
	δ-Elemene (12309449)	−6.4
	Germacrene D (5317570)	−7.1
	Bicyclogermacrene (13894537)	−7.6
	δ-Cadinene (441005)	−7.8
	Spathulenol (92231)	−7.2
	Caryophyllene oxide (1742210)	−7.4
	Fokienol (5352449)	−5.2
	Muurola-4,10(14)-dien-1-β-ol (6429100)	−7.0
	Sulcotrione (91760)	−7.6

**Table 4:** In silico ADME analysis of major constituents of Gsch and Xfru.

Entry	1	2	3	4	5	6	7	8	9	10
TPSA* (Å^2^)	0.00	0.00	0.00	0.00	0.00	0.00	20.23	12.53	20.23	20.23
Consensus * Log Po/w	3.44	3.42	4.49	4.30	4.15	4.12	3.26	3.68	4.72	3.37
Mol wt (g/mol)	136.23	136.23	204.35	204.35	204.35	204.35	220.35	220.35	248.4	220.35
nRB	0	0	3	1	0	1	0	0	9	1
nOHA	0	0	0	0	0	0	1	1	1	1
nWIND	0	0	0	0	0	0	1	0	1	1
WLOGP	3.00	3.00	4.75	4.89	4.73	4.73	3.26	3.94	4.95	3.70
Water solubility	Soluble	Soluble	Soluble	Soluble	Soluble	Soluble	Soluble	Soluble	Soluble	Soluble
GI absorption **	Low	Low	Low	Low	Low	Low	High	High	High	High
BBB permeant **	Yes	Yes	No	No	No	No	Yes	Yes	Yes	Yes
P-gp substrate **	No	No	No	No	No	No	No	No	No	No
CYP1A2 inhibitor **	No	No	No	No	No	No	No	No	Yes	No
CYP2C19 inhibitor **	No	No	Yes	Yes	Yes	Yes	Yes	Yes	No	Yes
CYP2C9 inhibitor **	Yes	Yes	Yes	Yes	Yes	Yes	No	Yes	Yes	No
CYP2D6 inhibitor **	No	No	No	No	No	No	No	No	No	No
CYP3A4 inhibitor **	No	No	No	No	No	No	No	No	No	No
Log *K_p_* (cm/s) (skin permeation)	−3.95	−4.18	−3.80	−4.18	−4.61	−4.85	−5.44	−5.12	−3.88	−5.48
Lipinski ***	Yes	Yes	Yes	Yes	Yes	Yes	Yes	Yes	Yes	Yes
Lipinski violation	1	1	1	1	1	1	0	0	1	0
Bioavailability score ***	0.55	0.55	0.55	0.55	0.55	0.55	0.55	0.55	0.55	0.55

ADMET: absorption, distribution, metabolism, excretion and toxicity, Lipophilicity *, Pharmacokinetics **, Drug likeliness ***, TPSA: topological polar surface area, nRB: no. of rotatable bonds, nOHA: no. of H-bond acceptors, nOHD: no. of H-bond donors, WLOGP: water partition coefficient, GI absorption: gastrointestinal absorption, BBB: blood–brain barrier, P-gp: glycoprotein permeability, CYP: cytochrome P450, Entry 1: α-pinene, 2: β-pinene, 3: δ-elemene, 4: germacrene D, 5: bicyclogermacrene, 6: δ-cadinene, 7: spathulenol, 8: caryophyllene oxide, 9: fokienol, 10: muurola-4,10(14)-dien-1-β-ol. Table 3. Toxicological properties of selected compounds from Gsch and Xfru.

**Table 5:** Toxicological properties of selected compounds from Gsch and Xfru.

Compounds	α-Pinene	β-Pinene	δ-Elemene	Germacrene D	Bicyclogermacrene D	δ-Cadinene	Spathulenol	Caryophyllene oxide	Fokienol	Muurola-4,10(14)-dien-1-β-ol
Hepatotoxicity	No	No	No	No	No	No	No	No	No	No
Carcinogenicity	No	No	No	No	No	No	No	No	No	No
Cytotoxicity	No	No	No	No	No	No	No	No	No	No
Immunotoxicity	No	No	No	Yes	No	No	No	Yes	No	No
Mutagenicity	No	No	No	No	No	No	No	No	No	No
Predicted LD_50_ (mg/kg)	3700	4700	5300	5300	5300	4390	3900	5000	5000	1016
Toxicity class	V	V	V	V	V	V	V	V	V	IV

Toxicity class: Class I: fatal if swallowed (LD50 ≤ 5), Class II: fatal if swallowed (5 < LD50 ≤ 50), Class III: toxic if swallowed (50 < LD50 ≤ 300), Class IV: harmful if swallowed (300 < LD50 ≤ 2000), Class V: may be harmful if swallowed (2000 < LD50 ≤ 5000), Class VI: nontoxic (LD50 > 5000).

## Data Availability

All data relating to the present study can be requested from the author for correspondence.

## Sample Availability

Samples of the compounds essential oil from *Guatteria schomburgkiana* Mart. and *Xylopia frutescens* Aubl. are available from the authors.
